# Mudança de Mentalidade na Doença Arterial Coronariana: Reflexões Desencadeadas pelo Sexagésimo Aniversário do Protocolo de Bruce

**DOI:** 10.36660/abc.20230638

**Published:** 2023-11-14

**Authors:** Nuno Bettencourt, Eduardo Vilela, António Miguel Ferreira

**Affiliations:** 1 Universidade do Porto Faculdade de Medicina Unidade de Investigação Cardiovascular Porto Portugal Universidade do Porto Faculdade de Medicina – Unidade de Investigação Cardiovascular, Porto – Portugal; 2 Serviço de Cardiologia Centro Hospitalar de Vila Nova de Gaia/Espinho Gaia Portugal Serviço de Cardiologia, Centro Hospitalar de Vila Nova de Gaia/Espinho, Gaia - Portugal; 3 Hospital Santa Cruz Centro Hospitalar de Lisboa Ocidental Hospital da Luz Lisboa Lisboa Portugal Hospital Santa Cruz – Centro Hospitalar de Lisboa Ocidental – Hospital da Luz Lisboa, Lisboa – Portugal

**Keywords:** Protocolo Bruce, Prova de Esforço, AngioTC Coronária, Testes de Isquemia, Carga Aterosclerótica

Seis décadas se passaram desde os estudos pioneiros que levaram ao desenvolvimento do protocolo de Bruce para o teste ergométrico, primeiro apresentado por Robert Arthur Bruce (o pai da cardiologia do exercício) et al.^1 , 2^ Esse marco no diagnóstico cardiovascular abriu caminhos para um melhor entendimento e manejo da Doença Arterial Coronariana (DAC). Enquanto comemoramos o sexagésimo aniversário do protocolo de Bruce, ainda existem incertezas acerca do melhor método de diagnóstico e manejo da DAC.^3 - 5^ De fato, o manejo da DAC sofreu uma importante mudança de paradigma nos últimos anos.^4 - 6^ O rápido avanço de modalidades invasivas e não invasivas (por exemplo, de imagens) possibilitou melhor desempenho diagnóstico e, assim, possíveis melhorias no manejo do paciente.^4 , 6^ Simultaneamente, vários ensaios clínicos desafiaram alguns dos conceitos fundamentais da DAC e geraram uma ampla discussão sobre a “mudança do paradigma da DAC”: do tratamento somente da estenose e isquemia da artéria coronária para uma abordagem mais ampla, envolvendo carga aterosclerótica total, estratificação de risco, e uma visão global do manejo do paciente.^4 - 8^ Essa evolução de pensamento ocorreu paralelamente ao desenvolvimento de ferramentas invasivas e não invasivas que nos permitiram estudar as múltiplas facetas da DAC.

## Do teste ergométrico clássico às modalidades modernas

O teste ergométrico, introduzido várias décadas atrás e cuja face mais visível evoluiu ao que se tornou o protocolo de Bruce, foi uma mudança de paradigma na avaliação da DAC.^1^ Ele possibilitou um meio não invasivo para avaliar a resposta do coração ao estresse físico, oferecendo *insights* valiosos para aspectos como alterações isquêmicas e arritmias.^1 , 4^ A simplicidade do método, combinada com o fornecimento de dados sobre diferentes componentes da resposta cardiovascular, levou à ampla utilização do teste ergométrico.^1^ No entanto, o cenário evolutivo da tecnologia médica introduziu métodos alternativos, tais como a ecocardiografia de estresse e imagem nuclear e, mais recentemente, técnicas de imagens avançadas de suporte, tais como ressonância magnética cardíaca e angiotomografia computadorizada (AC).^3 , 4 , 8^ De maneira lenta, mas constante, essas técnicas têm prevalecido em vários cenários, e diretrizes atuais atribuem um papel relativamente secundário para o teste ergométrico no diagnóstico de DAC, ao mesmo tempo em que destacam seu valor em inúmeros outros contextos, como na avaliação da capacidade funcional, de sintomas, do ritmo cardíaco e de distúrbios da condução, na prescrição de exercício e na cardiologia do esporte.^4 , 6 , 9 , 10^

## O enigma da escolha

Com uma vasta gama de modalidades disponíveis, médicos e pesquisadores agora encaram um enigma desafiador: qual abordagem oferece a informação mais precisa e prática em diferentes populações? Embora, historicamente, a angiografia coronária tenha sido descrita como o padrão ouro no diagnóstico de DAC, seu caráter invasivo e potenciais riscos, no entanto, requerem consideração cuidadosa.^4^ As técnicas de imagem não invasiva oferecem *insights* funcionais e anatômicos detalhados, mas sua disponibilidade e custos associados podem variar consideravelmente.^3 , 9 - 12^ Esse cenário diverso de potenciais escolhas demanda uma abordagem individualizada de cada paciente, integrando fatores como apresentação clínica, perfil de risco, comorbidades, preferências do paciente, e recursos disponíveis.^4 , 6 , 8 - 10^ Além disso, entre os fatores mais importantes (embora muitas vezes esquecidos) na análise de um teste, está o equilíbrio risco-benefício e seu potencial impacto sobre as estratégias de tratamento pré-teste. Um ponto importante, caso não seja esperado que o manejo do paciente mude independentemente dos resultados do teste, então, deve-se reconhecer que muito provavelmente o teste não é apropriado.^4 , 10^

## Além da estenose da artéria coronária: abrangendo a carga aterosclerótica

Tradicionalmente, a avaliação e o manejo da DAC giraram em torno da identificação e do tratamento de estenoses importantes da artéria coronária.^8^ Contudo, pesquisas têm progressivamente revelado as limitações dessa abordagem.^5 , 8 , 9^ De fato, a ausência de placas obstrutivas na angiografia (ou seja, na angiografia coronária invasiva) não exclui a presença de anormalidades na função vascular coronária.^6 , 9^ Esse conceito, associado não só a distúrbios na microcirculação e à isquemia com DAC não obstrutiva (entidade cada vez mais reconhecida), como também a outras condições como a angina vasoespática, deve ser considerada na avaliação de indivíduos com dor torácica.^4 , 6 , 9^ Por outro lado, nem todas as lesões estenóticas levam a eventos adversos, e algumas placas não obstrutivas podem ser vulneráveis e tender a se romperem, como mostrado elegantemente em indivíduos após uma síndrome coronária aguda.^3 , 13^ Consequentemente, um foco na carga aterosclerótica – a extensão e a distribuição da placa dentro das artérias coronárias – surgiu como um indicador mais abrangente do risco cardiovascular.^3 , 9^ Modalidades de imagem como o escore de cálcio coronariano, AC e imagem intravascular empoderaram médicos a quantificar a carga aterosclerótica, possibilitando uma estratificação de risco e decisões terapêuticas mais precisas e personalizadas.^4 , 8 , 9^ Com uma identificação precoce dos pacientes em alto risco, os recursos podem ser alocados de maneira eficiente, e estratégias preventivas podem ser otimizadas.^3 , 9 , 14 , 15^

Enquanto a troca de paradigma no entendimento atual sobre a fisiopatologia e o manejo da DAC é promissor na tentativa de melhorar os desfechos do paciente, ela também apresenta desafios significativos.^3 , 4 , 14^ Como e com que frequência nós devemos quantificar a carga aterosclerótica, quais limiares e intervenções devemos usar, são algumas das questões imediatas que permanecem não respondidas. Mas, talvez o maior desafio em implementar essa troca de paradigma seja a transformação de mentalidade que será necessária em diferentes clínicas de cardiologia e departamentos. Colaboração interdisciplinar, educação continuada, e integração da medicina baseada em evidência na prática clínica são necessárias para se alcançar o potencial máximo desse novo paradigma.

## Olhando adiante

A mudança de paradigma mencionada acima representa um momento crucial na área multifacetada e dinâmica da DAC. Enquanto necessidades não atendidas quanto à melhor abordagem em diferentes cenários persistem (particularmente quando são consideradas as especificidades de cada paciente), evidências têm desafiado médicos a ampliarem suas perspectivas e se moverem em direção a uma medicina mais centrada no paciente.^3 , 4 , 6 , 9 , 10 , 14^ Ao comemorar o aniversário de 60 anos do teste ergométrico com o protocolo de Bruce, vamos aproveitar a oportunidade de reavaliar pragmaticamente nossas estratégias para o diagnóstico e o manejo da DAC. Mudando nosso foco de lesões individuais para a carga aterosclerótica, ao implementar modelos de estratificação de risco personalizados e adotar uma abordagem holística do paciente, estamos promovendo uma nova era de tratamento da DAC, que pode ser mais precisa, abrangente, e eficaz que já houve antes. Apesar de ainda existirem várias questões que necessitem mais estudos em termos de seleção de um melhor teste em diferentes pontos do *continuum* da DAC, o sexagésimo aniversário do protocolo de Bruce é um lembrete do grande progresso feito no diagnóstico cardiovascular e no entendimento de sua complexa patologia.^3 , 4 , 6 , 8^ Nosso desafio atual como médicos é trazer todo esse conhecimento acumulado para a prática clínica e adotar estratégias baseadas em evidência para o diagnóstico e tratamento da DAC, mesmo se isso represente uma profunda mudança no paradigma que estamos historicamente seguindo.
