# Supplementary material for: Mudança de Mentalidade na Doença Arterial Coronariana: Reflexões Desencadeadas pelo Sexagésimo Aniversário do Protocolo de Bruce
Source: Arq Bras Cardiol. 2023 Nov 14;120(10):e20230638. [Article in Portuguese] doi: 10.36660/abc.20230638 (PMC10697679; doi:10.36660/abc.20230638)
Supplement: Translation

# Translation: Mindset Shift in Coronary Artery Disease: Reflections Triggered by the Diamond Anniversary of the Bruce Protocol

**Authors:** Nuno Bettencourt, Eduardo Vilela, António Miguel Ferreira

**Affiliations:** 1 Portugal Universidade do Porto Faculdade de Medicina – Unidade de Investigação Cardiovascular, Porto – Portugal; 2 Portugal Serviço de Cardiologia, Centro Hospitalar de Vila Nova de Gaia/Espinho, Gaia - Portugal; 3 Portugal Hospital Santa Cruz – Centro Hospitalar de Lisboa Ocidental – Hospital da Luz Lisboa, Lisboa – Portugal

**Keywords:** Bruce Protocol, Treadmill Test, Coronary CT, Ischemia Testing, Atherosclerotic Burden

Six decades have passed since the pioneering studies that led to the Bruce protocol for exercise electrocardiogram (ECG) testing, first presented by Robert Arthur Bruce (the father of exercise cardiology) et al.^1 , 2^This landmark in cardiovascular diagnostics paved the way for a better understanding and management of coronary artery disease (CAD). While we celebrate the 60^th^anniversary of the Bruce protocol, ongoing uncertainties regarding the best way to diagnose and manage CAD remain.^3 - 5^Indeed, CAD management has undergone a profound paradigm shift over recent years.^4 - 6^The rapid advancement of invasive and non-invasive (i.e. imaging) modalities has allowed better diagnostic performance and hence possible improvements in patient management.^4 , 6^Simultaneously, several clinical trials have challenged some of the fundamental concepts of CAD and prompted a full discussion concerning the “CAD paradigm shift”: from treating coronary artery stenosis and ischemia only to a broader approach encompassing overall atherosclerotic burden, risk stratification, and a global perspective on patient management.^4 - 8^This evolution in thinking has paralleled the development of invasive and non-invasive tools which enable us to study the multiple facets of CAD.

## From classical ECG exercise testing to modern modalities

The ECG exercise test, introduced several decades ago and whose most visible face was evolved to what would become the Bruce protocol, was a paradigm shift in CAD evaluation.^1^It provided a non-invasive means to assess the heart’s response to physical stress, offering valuable insights into aspects like ischemic changes and arrhythmias.^1 , 4^The simplicity of this method, coupled with the provision of data on different components of the cardiovascular response, led to its widespread adoption.^1^However, the evolving landscape of medical technology has introduced alternative methods such as stress echocardiography and nuclear imaging and, more recently, ancillary advanced imaging techniques such as cardiac magnetic resonance imaging and computed tomography angiography (CTA).^3 , 4 , 8^Slowly but steadily these techniques have prevailed in several scenarios, and current guidelines reserve a somewhat secondary role for ECG exercise testing in the diagnosis of CAD. Meanwhile, they highlight its value in a plethora of other clinical contexts, namely in terms of assessment of functional capacity, symptoms, heart rhythm and conduction disturbances, exercise prescription, and sports cardiology.^4 , 6 , 9 , 10^

## The conundrum of choice

With a vast array of available modalities, clinicians and researchers now face a challenging conundrum: which approach offers the most accurate and actionable information in different patient populations? Although invasive coronary angiography has historically been described as the gold standard for diagnosing CAD, its invasiveness and potential risks nonetheless warrant careful consideration.^4^Non-invasive imaging techniques offer detailed anatomical and functional insights, but their availability and associated costs can vary widely.^3 , 9 - 12^This diverse landscape of potential choices demands an individualized approach for each patient, integrating factors such as clinical presentation, risk profile, comorbidities, patient preferences, and available resources.^4 , 6 , 8 - 10^Additionally, among the most relevant (yet often forgotten) factors to be considered in a test is its risk-benefit balance and potential impact on pre-test treatment strategies. Importantly, if patient management is not expected to change independently of the test results, then it should be acknowledged that the test is probably inappropriate.^4 , 10^

## Beyond coronary artery stenosis: embracing atherosclerotic burden

Traditionally, the assessment and management of CAD has revolved around identifying and treating significant coronary artery stenosis.^8^However, research has progressively revealed the limitations of this approach.^5 , 8 , 9^Indeed, the absence of obstructive plaque on angiography ( *i.e* ., in the setting of invasive coronary angiography) does not exclude the presence of abnormalities in coronary vascular function.^6 , 9^This, associated not only with derangements in the microcirculation and the increasingly recognized entity of ischemia with non-obstructive CAD, but also with other conditions such as vasospastic angina, should be considered when assessing individuals with chest pain.^4 , 6 , 9^On the other hand, not all stenotic lesions lead to adverse outcomes, while some non-obstructive plaques may be vulnerable and prone to rupture, as elegantly shown in individuals after an acute coronary syndrome.^3 , 13^Consequently, a focus on atherosclerotic burden - the extent and distribution of plaque within the entire coronary tree - has emerged as a more comprehensive indicator of cardiovascular risk.^3 , 9^Imaging modalities such as coronary calcium scoring, CTA and intravascular imaging have empowered clinicians to quantify atherosclerotic burden, enabling more accurate and personalized risk stratification and treatment decisions.^4 , 8 , 9^By identifying high-risk patients early, resources may be allocated efficiently, and preventive strategies could be optimized.^3 , 9 , 14 , 15^

While the paradigm shift in the current understanding of CAD physiopathology and management is promising in the quest to improve patient outcomes, it also presents significant challenges.^3 , 4 , 14^How and how often we should quantify atherosclerotic burden, which thresholds and which interventions we should use, are some of the pressing questions that remain largely unanswered. But perhaps the greatest challenge in implementing this paradigm shift will be the mindset transformation that will be required across different cardiology clinics and departments. Interdisciplinary collaboration, continuous education, and the integration of evidence-based medicine into clinical practice is necessary to reach the full potential of this new paradigm.

## Looking forward

The abovementioned paradigm shift represents a pivotal moment in the multifaceted and dynamic field of CAD. While unmet needs persist regarding the optimal approach in distinct scenarios (particularly when considering the specificities of each patient), evidence has challenged clinicians to broaden perspectives and move towards a more patient-centered medicine.^3 , 4 , 6 , 9 , 10 , 14^As we celebrate the diamond anniversary of ECG exercise testing with the Bruce protocol, let us embrace the opportunity to pragmatically reevaluate our strategies for CAD testing and management. By shifting our focus from individual lesions to atherosclerotic burden, while implementing personalized risk stratification models and adopting a holistic patient approach, we are promoting a new era of CAD care that can be more precise, comprehensive, and effective than ever before. Although there are still several issues which need further study in terms of optimized test selection across different points of the CAD continuum, this 60^th^anniversary of the Bruce protocol is a reminder of the remarkable progress made in cardiovascular diagnostics and in the understanding of this complex pathology.^3 , 4 , 6 , 8^Our current challenge as physicians is to bring this cumulative body of knowledge into clinical practice and adopt evidence-based strategies for the diagnosis and treatment of CAD, even if this represents a profound change in the paradigm that we have historically followed.
